# Polyaniline: Doping and Functionalization with Single Walled Carbon Nanotubes for Photovoltaic and Photocatalytic Application

**DOI:** 10.3390/polym13162595

**Published:** 2021-08-05

**Authors:** Mariem Saoudi, Boubaker Zaidi, Abdullah A. Alotaibi, M.G. Althobaiti, Eid M. Alosime, Ridha Ajjel

**Affiliations:** 1 Laboratory of Energies and Materials (LabEM), Higher School of Sciences and Technology of Hammam Sousse, Sousse University, Sousse 4011, Tunisia; mariem_saoudi@yahoo.fr (M.S.); ajjelridha@gmail.com (R.A.); 2 Laboratoire Physico-Chimie des Matériaux, Département de Physique, Faculté des Sciences de Monastir, Université de Monastir, Monastir 5000, Tunisia; 3 Department of Physics, Faculty of Science, Shaqra University, Dawadmi 11911, Saudi Arabia; 4 Department of Chemistry, Faculty of Science, Shaqra University, Dawadmi 11911, Saudi Arabia; aaalotaibi@su.edu.sa; 5 Department of Physics, Faculty of Science, Taif University, Taif 888, Saudi Arabia; m.althobaiti@tu.edu.sa; 6 King Abdulaziz City for Science and Technology (KACST), P.O. Box 6086, Riyadh 11442, Saudi Arabia; alosimi@kacst.edu.sa

**Keywords:** doping, carbon nanotubes, functionalization, optical characterization, DFT, solar cells

## Abstract

Polyaniline (PANI) was chemically doped and functionalized with single walled carbon nanotubes (SWCNTs). Various characterization methods were employed to study the structure and optical properties of PANI/SWCNTs nanocomposite, such as Fourier transform infrared (FTIR), differential scanning calorimetry (DSC), scanning electron microscopy (SEM), optical absorption, and stationary photoluminescence. Additionally, a theoretical study using density functional theory calculations was also carried out. It has been demonstrated that the doping process may reduce the band gap without affecting the molecular structure, leading to a better compatibility with the solar spectrum. Moreover, the functionalization process with SWCNTs was able to significantly improve the properties of the resulting nanocomposite. The final interpenetrating network of PANI/SWCNTs exhibited an optical gap of nearly 2.28 eV, from which localized states induced by the charge transfer were created at nearly 1.70 eV. In addition, the resulting donor–acceptor network leads to a separation of electron holes pairs rather than their recombination, which can be used as an active layer in photovoltaic applications and a photocatalyst for advanced oxidation processes.

## 1. Introduction

A significant number of works have been focused on exploiting the properties of nanoparticles [1]. New nano-structured materials with improved properties can be elaborated by inserting nanoparticles into the organic matrix [2,3]. Since the 1990s, considerable efforts have been devoted for carbon nanotubes (CNTs), due to their form factor and their unique and reproducible properties. The electronics industry is currently developed around organic materials [4,5,6] in an attempt to replace silicon technology with lower cost and easier processing. Polymer materials are widely studied as active layers for electronic devices where the majority of handicaps are related to their fragility and their limited operation lifetime. The addition of the small quantities of CNTs permits not only the improvement of their mechanical properties [7], but also offers a higher operating life time [8]. Particularly, the good dispersion of CNTs in the polymer matrix leads to charge transfer and good transport properties due to their high electron and hole mobilities [9]. Physically, charge transfer is created by nano-junctions imposed by interpenetration of the donor and acceptor domains [10]. In addition, the resulting interpenetrating network may improve the electron–hole pairs after photo-excitation, leading to a good collection process. The obtained bulk hetero-nano-junctions lead to high photovoltaic conversion efficiency [11,12]. Among the conducting polymers, PANI is the most studied due to its physical and chemical properties that can be easily modified [13,14]. Particularly, PANI exhibits better environmental stability and easy synthesis [15,16]. In this context, a simple treatment of PANI results in a new degree of oxidation where its conductivity can be reversibly switched from the insulating to the conductive state [17]. Recently, it has been demonstrated that PANI/SWCNTs composites gave enhanced field emission characteristics due to π–π interaction between the quinoid rings of PANI and the π bond of the SWCNTs lattice [18]. Moreover, the nanocomposite exhibits significant enhancement in both electrical and thermoelectric properties [19]. On the other hand, the modified PANI/SWCNTs nanocomposite was used for the electrochemical determination of some metals with good selectivity and sensitivity [20]. As the leucomeraldine base form is generally an insulator, it will be useful to carry out a doping process before its functionalization with the CNTs [21]. The procedure of acid doping [22,23] is the easiest method and leads to switching between its oxidized forms.

In order to establish a good structure property correlation, experimental conclusions are often supported by theoretical methods [24,25]. Quantum calculations based on functional density theory (DFT) are the most appropriate tools to describe organic materials’ properties [26,27]. Using these calculations, we can predict properties of materials and their corresponding application field [27,28]. Due to the development of computational processors, it is currently possible to carry out calculations using higher degrees of freedom with a higher basis set of computation [26,27].

In this paper, we are primarily interested in establishing a structure–property correlation by means of experimental and theoretical characterization techniques of both doped and pure PANI states. We also investigate and describe the functionalization of PANI with SWCNTs, particularly the nature of the resulting nano-junctions at the molecular scale. Fourier transform infrared (FTIR), differential scanning calorimetry (DSC) and scanning electron microscopy (SEM) techniques are used to evidence changes in vibrational and structural properties. However, changes in the optical properties are evaluated by means of optical absorption and photoluminescence spectroscopies. The obtained results are correlated with those obtained theoretically using the DFT.

## 2. Materials and Methods

PANI and SWCNTs were produced by Sigma Aldrich (St. Quentin Fallavier, Cedex France). At 300 K, the black colored PANI powder was characterized by a purity of 99.99%, an average macromolecular mass Mn > 15,000, a refractive index of 1.85, a melting temperature above 600 K, and a density of 1.36 g/mL. SWCNTs were obtained by the electric arc technique where the diameter of the individual tube varied from 1.0 to 1.2 nm, while they exhibited a typical length of 500 nm [26,29]. The PANI doping process was similar to the classical method reported by A. G. MacDiarmid et al. [23]. The process was accomplished in a solution where the PANI was placed for two hours in a mixture of 20% sulfonic acid (R-SO_3_H) and 80% dimethyl formamide (DMF). After evaporation of the solvent and drying, the obtained product was washed three times with DMF to remove residual acid particles. The functionalization process of SWCNTs with PANI is a purely mechanical method [30]. SWCNTs’ are dispersed in the appropriate solvent (DMF) in order to isolate bundled tubes, using the velocity of 8000 rpm for three hours. The obtained composite is then introduced into an ultrasonic bath and submitted to centrifugal forces for 30 min in order to homogenize SWCNTs’ distribution and orientation in the PANI matrix [31,32,33]. The viscous obtained solutions were deposited with nearly uniform thickness on glass slides for FTIR and photoluminescence measurements and on silica slides for optical absorption measurements. All substrates were already cleaned with deionized water and ethanol in an ultrasonic bath. FTIR measurements were conducted using a Bruker Vertox 80 V interferometer (Bruker Optics, Ettlingen, Germany) with a resolution of 4 cm^−1^. The DSC measurements were recorded on a Perkin Elmer DSC 8500 analyzer (Perkin Elmer, Inc. Shelton, CT, USA). All samples in the powder state had nearly the same weight of 2.6 mg. During measurements, the temperature was scanned from 323 K to 600 K, at the heating rate of 10 K/min, under nitrogen atmosphere, for which the Gas Switch to nitrogen was 20 mL/min. The optical absorption spectra were recorded using a UV1800 spectrophotometer (Shimadzu Scientific istruments, Inc. Colombia, MD, USA) working in the absorption mode with the wavelength varying from 200 nm (6.2 eV) to 2000 nm (0.62 eV). The SEM micrographs were carried out using the JEOL 7600 F microscope (JOEL, Inc, Peabody, MA, USA) which was equipped with a source delivering field effect, operating with the voltage of 10 KV, making it possible to obtain electron beams of a very large smoothness, reaching a space resolution of a few tens of nanometers. For photoluminescence (University of Monastir, Tunisia), in order to cover the entire emission spectrum, two excitation wavelengths were used (325 nm and 540 nm). Theoretical data were obtained from quantum calculations based on density functional theory (DFT), the most appropriate tool to describe the organic materials’ properties [27,34]. The modeling structure has been fully optimized using the most popular three-parameter hybrid function, Becke’s B3 [35], with the basis set 6-31-G (d). Calculations applied to the modeling structure were conducted with the non-local correlation of Lee–Yang–Parr, LYP, abbreviated as the B3LYP method [36], without constraint, which was often used in in our previous calculations [37]. Optical absorption spectra were calculated using the time-dependent density functional theory (TD-DFT) method with the 6-31G (d) basis set and were fitted into Gaussian curves within the Swizard program [27,37].

## 3. Results and Discussion

**Figure 1:**
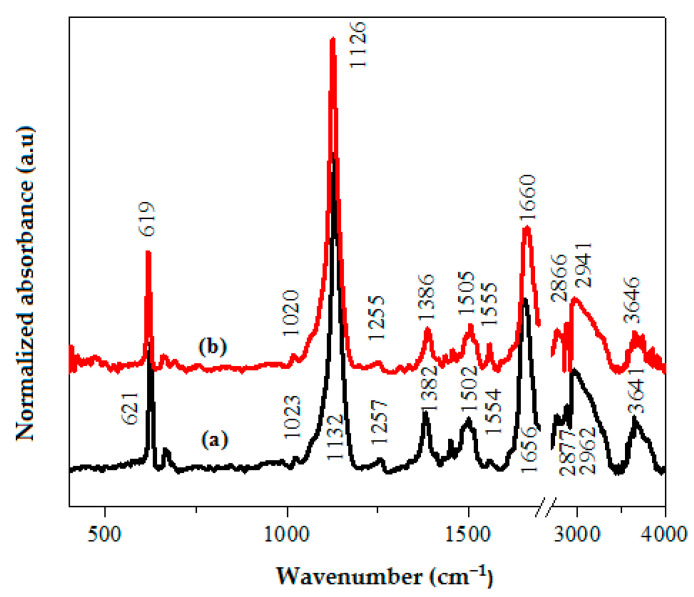
FTIR spectrum of pure (**a**) and acid-doped (**b**) PANI.

Figure 1 shows the infrared absorption spectra of the PANI in both the pure and acid-doped states. Band positions and their assignments are summarized in Table 1 [30,38].

**Scheme 1:**
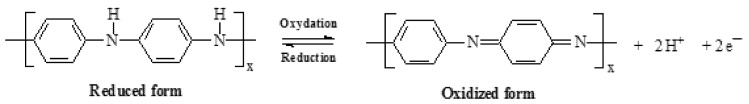
Mechanism of acid doping of the leucomeraldine base form: transformation to the oxidized form (EB).

The spectra of Figure 1 have been normalized by referring to the band situated at 1132 (1126) cm^−1^, attributed to the benzene ring deformation. Spectra show that the same bands are found after doping but the band intensity and positions are changed (Table 1), demonstrating that the material skeleton is conserved. The intensity increase/decrease and the slight frequency shift are the consequences of the geometrical relaxation after doping. Particularly, the band at 1554 cm^−1^, attributed to the C=C vibration of the quinoid form, has been greatly intensified. At the same time, the band at 1656 cm^−1^, attributed to the same group but relative to the aromatic form, is decreased. This demonstrates that the undoped leucomeraldine form is partially transformed into its oxidized form, inducing the appearance of a local charge (Scheme 1). This local charge formation is the consequence of hydrogen departure, leading to a new oxidation degree [23].

**Figure 2:**
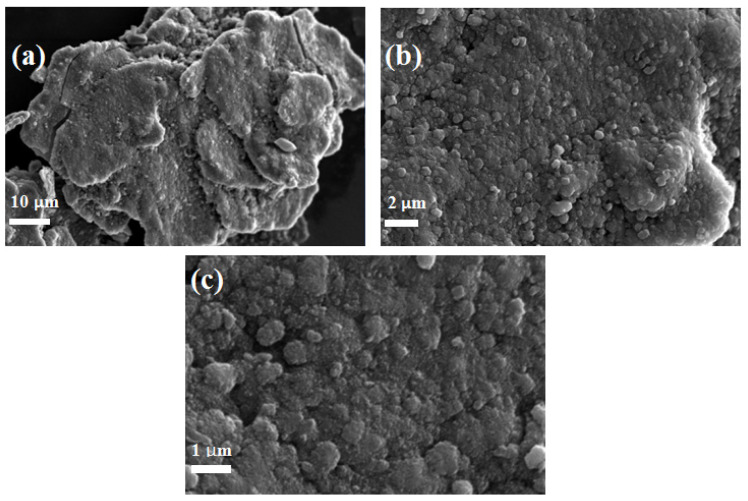
SEM images of (**a**,**b**) PANI and (**c**) sulfonic acid-doped PANI.

Figure 2 shows SEM pictures of PANI in both the pure and doped states. It is clearly seen that the structure of undoped PANI is constituted by agglomerations with sizes of a few tens of microns (20 to 30 microns). These agglomerations are composed of granular features with 1–2 µm diameters, comparable to those commonly found in the literature [39,40,41]. We also note that after doping, the PANI structure still remains granular where the grain size has been decreased (ranging from 0.5 to 1 μm). We believe that the doping species, which is a strong acid, leads to the increase in the solubility of PANI and, consequently, the decrease in grain size. 

**Figure 3:**
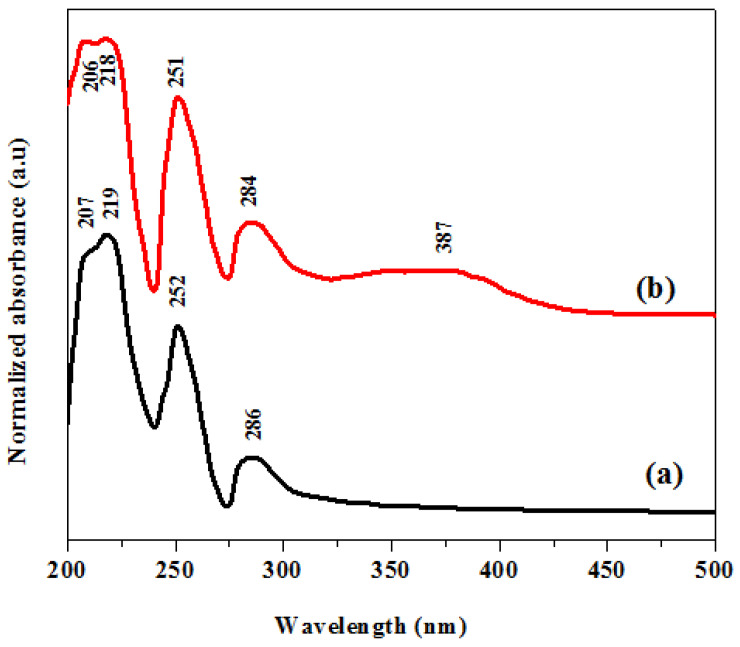
Optical absorption spectra of PANI in the pure (**a**) and doped (**b**) states.

Figure 3 shows the changes in the optical absorption spectrum after the PANI sulfonic acid doping. It should be noted that the absorption spectrum of pure PANI exhibits four absorption bands situated at 207 nm, 219 nm, 252 nm and 286 nm. According to the literature [42], these bands are, respectively, attributed to σ–σ*, σ–π *, π–σ* and π–π* transitions. After acid doping, the absorption spectrum is strongly affected. Principally, we show the creation of a new absorption band at 387 nm, supporting the optical gap reduction. This broad band is the consequence of the transformation of PANI from leucomeraldine form to the emeraldine salt/base forms [43], which agrees well with FTIR results. Therefore, the newly created optical transition is the consequence of a new energetic level in the PANI band gap [44]. A similar effect has been already observed for HCl-doped PANI [45].

**Figure 4:**
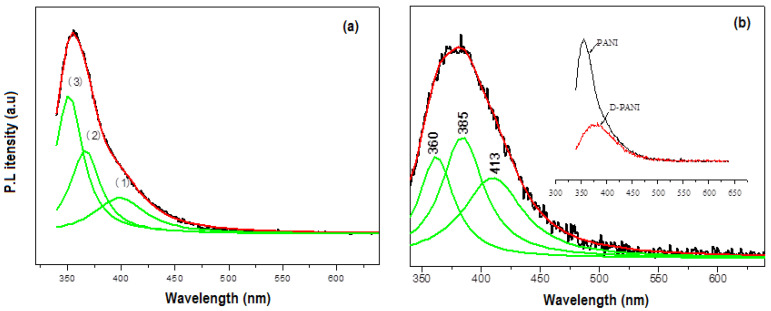
Photoluminescence spectra of pure (**a**) and sulfonic acid-doped (**b**) PANI. Black line: experimental; red line: Gaussian fit; green line: Gaussian components. The inset shows the PL intensity decrease from pure to doped PANI.

Photoluminescence spectra of Figure 4 show that the PANI emits in the spectral range from 340 nm to 480 nm, similar to the previously reported results [46]. The decomposition of PL spectrum shows three lines separated by the same amount of energy (in the order of 215 meV). As this energy corresponds approximately to the most intense band observed on the FTIR spectrum, at approximately 1660 cm^−1^ [47], we believe that these lines are derived from 0–0, 0–1 and 0–2 phonon emission. The acid doping is accompanied by a PL quenching effect, indicating that quinoidal fragments are created, representing inter-channel energy dissipation [48]. On the other hand, the spectrum has been red shifted by nearly 14 nm, showing that there is a sufficient decrease in the optical gap.

As it is often generalized, the optical gap can be elucidated from the variation of the absorption coefficient as a function of energy, according to the Tauc equation of [49], as shown in Equation (1) below:

(1) $$\alpha h\nu =B{\left(h\nu -\mathrm{Eg}\right)}^{n}$$

**Figure 5:**
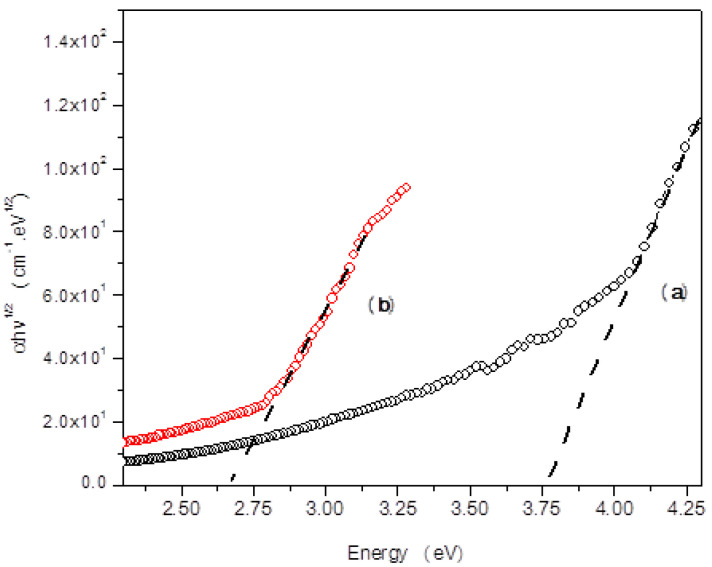
Variation of (αhν)^1/2^ versus hν for pure (**a**) and sulfonic acid-doped (**b**) PANI.

In this expression, α represents the absorption coefficient, B is a band tail parameter, Eg is the band gap of the material and n is an index representing the transition probability, taken equal to 2 for indirect transitions in amorphous materials [45,50]. The variation of (αhν)^1/n^ as a function of hν (Figure 5) makes it possible to give the value of the gap, which is estimated by extrapolation of the linear part occurring in the absorption threshold [45,51]. The band gap relative to the pure PANI evaluated to 3.75 eV decreases, reaching 2.63 eV after doping. These values are comparable to those previously published for both the pure and doped PANI forms [45]. In fact, for low photon energy, the spectral dependence of the absorption coefficient (α) as a function of energy (hν) is known as Urbach’s empirical law, in accordance with Equation (2) [52]:

(2) $$\alpha =\alpha_{0}\exp(\frac{h\nu }{E_{U}})$$

**Figure 6:**
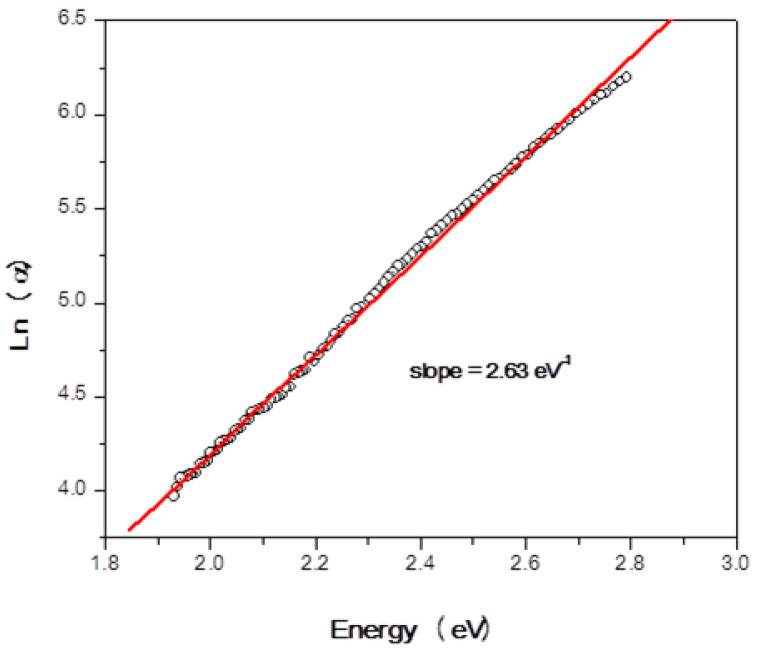
Variation of Ln (α) as a function of the photon energy in the case of doped PANI. (o) Experimental, (—) linear fit.

In this expression, α_0_ is a constant and E_U_ is the band-tail energy that is commonly referred to as Urbach energy [52]. Generally, this energy is weakly temperature dependent and is often interpreted as the width of localized states, created within the optical gap. These states are associated with disorder for amorphous and low crystalline materials [52,53]. Therefore, the variation of Ln (α) as a function of the photon energy presents linearity behavior in the concerned spectral range. In this case, the Urbach energy e (E_U_) can be obtained from the slope shown in Figure 6.

(3) $$\mathrm{Ln}\;\left(\alpha \right)=\mathrm{Ln}\;\left(\alpha_{0}\right)+\frac{h\nu }{E_{U}}$$

From Figure 6, we estimate the E_U_ in the order of 380 meV involved by the broad absorption band created within the PANI band gap. According to the literature, if the doping rate is high, the doped materials exhibit a continuum of individual levels [54]. Their recovery leads to a large band, as is observed in our case. This result agrees well with prior studies for which the doping procedure was carried out at saturation (higher doping level) [55].

**Figure 7:**
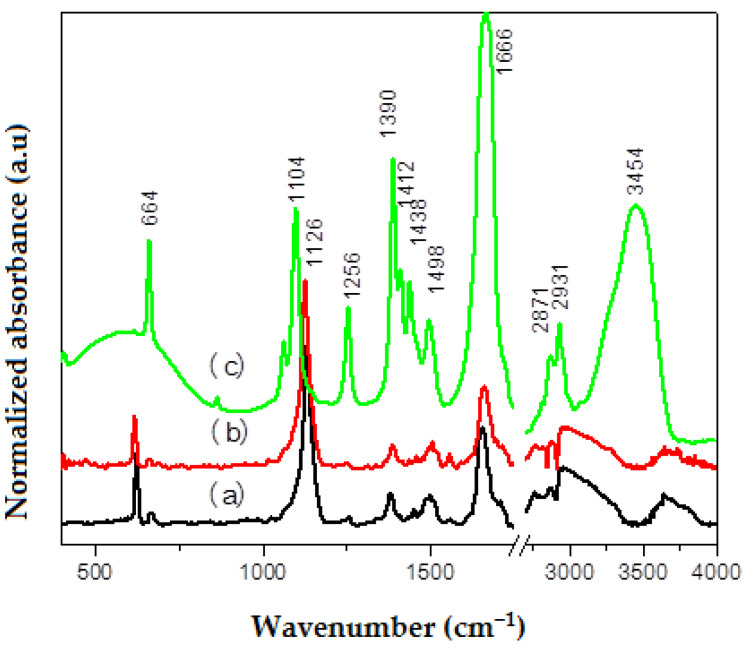
FTIR spectra of PANI (**a**), doped PANI (**b**) and Doped PANI/SWCNTs (**c**).

To evidence changes on the molecular structure after SWCNTs’ functionalization process, the obtained composite was studied by FTIR spectroscopy. In Figure 7, we represent the FTIR spectrum of the 2.10% SWCNTs weight concentration nano-composite with those of the PANI in both the pure and doped states. 

The decision to use this relatively lower weight concentration for SWCNTs was principally made with the aim of reaching a good dispersion process. Compared to the doped PANI, the spectrum shows the creation of new intense absorption bands at 1256, 1412 and 1438 cm^−1^. These new bands clearly represent the interaction between both components of the composite. The functionalization process is governed by a grafting reaction between PANI and carbon nanotubes. In this context, the new vibrational frequencies reflect vibration modes of SWCNTs and/or those relative to the linking between both constituents of the composite in the new chemical environment [10,56]. The modes of some frequencies, such as 664 cm^−1^ and 1126 cm^−1^, are shifted. This shift reflects new molecular arrangement due to the insertion of SWCNTs on the organic matrix. Similar effects were observed for other CNT-based composites [57]. 

**Figure 8:**
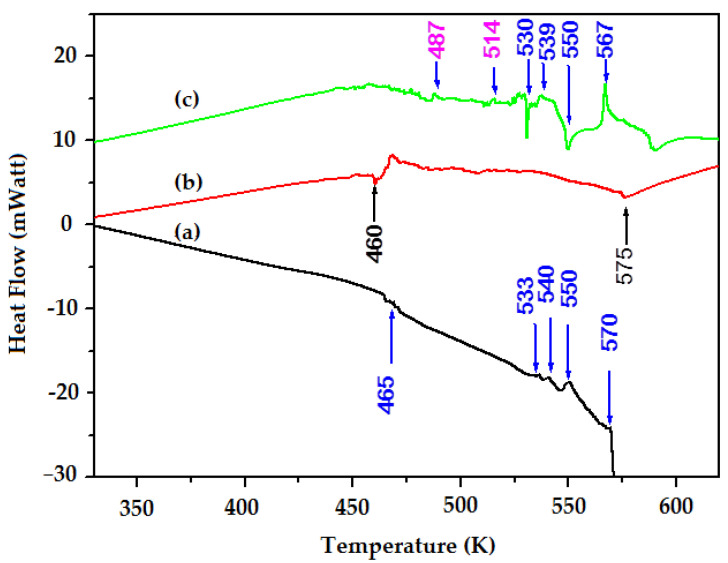
DSC thermograms of D-PANI (**a**), of SWCNTs (**b**) and of D-PANI/SWCNTs composite (**c**).

To provide additional information on the functionalization process between D-PANI and SWCNTs, we present, in Figure 8, DSC thermograms of either doped PANI, SWCNTs or the resulting nano-composite.

Compared to the PANI with thermal stability for temperatures lower than 465 K, the nano-composite results in much higher thermal stability, as is reported for other PANI-based nano-composites [58]. As a result, if there is no interaction between both components, the final structure will be a mixture of non-interacting compounds and the corresponding DSC thermogram will certainly be the superposition of individual thermograms. This hypothesis is not the case of Figure 8c, since the nano-composite exhibits additional thermal transitions in comparison to the D-PANI. These additional thermal transitions peaked at 487 K and 514 K, and are also not found in the case of SWCNTs. We think, therefore, that these latter transitions are the consequence of new structural modifications, mainly the de-grafting process between both components. For SWCNTs, the first thermal stage is observed in the temperature range of 460–470 K, for which there are two apparent thermal transitions. We think that these transitions are a consequence of the tube defect departure; they are not envisaged in the case of the nano-composite, proving that these defects are removed under the functionalization process. The browed peak starting from 575 K has been attributed to the wall degradation, typically the breaking of the SWCNTs’ C-C bond. For the PANI, added to the melting point at temperatures higher than 570 K, the DSC thermogram shows four principal thermal transitions centered, respectively, at 465 K, at 533 K, at 540 K and at 550 K. According to the previously published results [59], the first and the fourth ones are, respectively, related to the defect in D-PANI (residual doping agents) and the beginning of PANI degradation. However, both narrow intermediate peaks are, in our opinion, attributed to the ring opening and chain scission resulting from the already cross-linked chains under temperature elevation, as suggested in the case of PANI polymers [60,61]. The same behavior has been also evidenced in our previous work for annealed PANI/SWCNTs composite [62].

**Figure 9:**
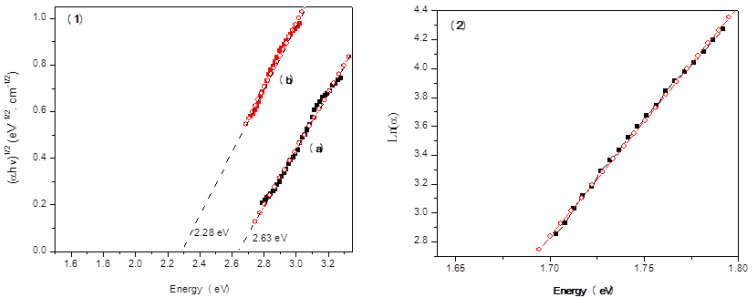
(**1**): Variation of the optical gap (a) sulfonic acid-doped PANI and doped PANI functionalized with SWCNTs (**2**): Evaluation of the localized states’ parameters (■): experimental. (o): linear fit.

The inserting of SWCNTs on the PANI matrix generally results in the creation of a charge transfer [63]. If there is compatibility between the junction size and the diffusion length, the created photo-excitation in the polymer matrix can reach the carbon nanotubes after the diffusion process [64,65]. In this context, the changes of optical absorption spectra after the insertion of the SWCNTs in the doped PANI are already published in our previous work [29]. The spectrum shows that the SWCNTs’ functionalization process is accompanied by a broadening effect of the already created band caused by doping, and a new absorption band appears at around 572 nm. The observed feature at lower energy is due to the creation of localized states [45]. To elucidate the quantitative aspect of these effects, we present, in Figure 9, the optical gap variation and localized states’ characteristics using, respectively, Tauc and Urbach relations [49,52] (Equations (1) and (2)).

The optical gap after doped PANI is reduced to 2.63 eV, showing energy levels creation within the band gap. These levels are associated with polaronic and/or bipolaronic species [42]. However, after adding SWCNTs, the latter is more decreased, reaching the value of 2.28 eV. Moreover, new localized states are also created at 1.62 eV, with an Urbach energy (E_U_) of 80 meV. These states often originate from the charge transfer between PANI and SWCNTs.

**Figure 10:**
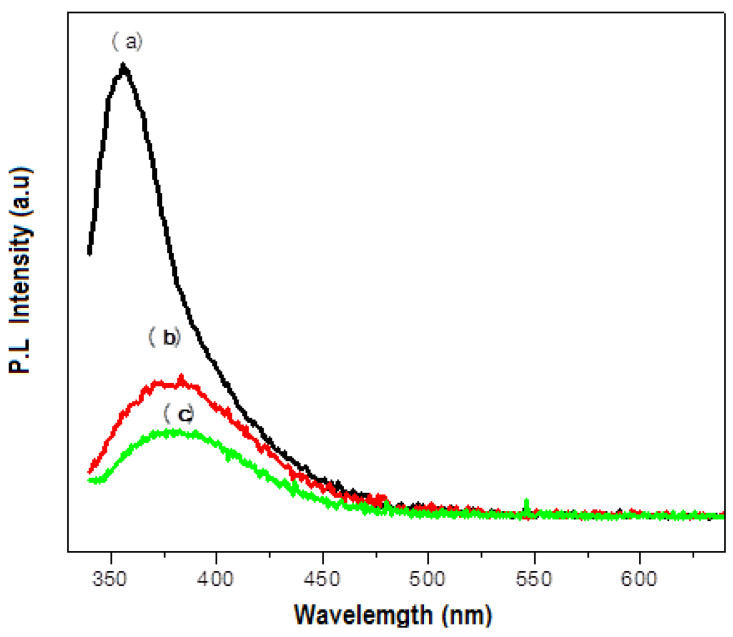
Photoluminescence spectra of (**a**) PANI, (**b**) doped PANI and doped-PANI/SWCNTs composite at 2.1% concentration (**c**).

The effect of the addition of SWCNTs is also evidenced by photoluminescence spectrum variation (Figure 10). The spectra show that, independently of the PANI state, (pure, doped or functionalized with SWCNTs), there is emission in the spectral domain ranging from 350 to 500 nm. Then, it is clearly seen that the insertion of SWCNTs on the doped PANI leads to quenching effects. This effect is often envisaged for CNT-based polymer composites and also in the case of the acid doping process [66,67]. Otherwise, the spectrum shows a slight shift towards the red region and a new narrow feature at approximately 550 nm. This weak luminescent center corresponds to the already created localized state due to the charge transfer. The weak intensity of this peak implies that a separation process has occurred rather than a recombination process for these transferred charges. As the PANI/SWCNTs composite absorbance exhibits a good compatibility with the solar spectrum, it can serve as an active layer for organic photovoltaic solar cells and a photocatalyst for advanced oxidation processes under solar irradiation. 

**Scheme 2:**
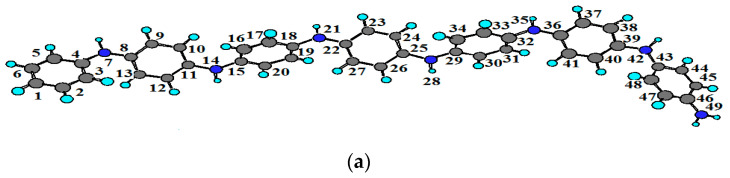

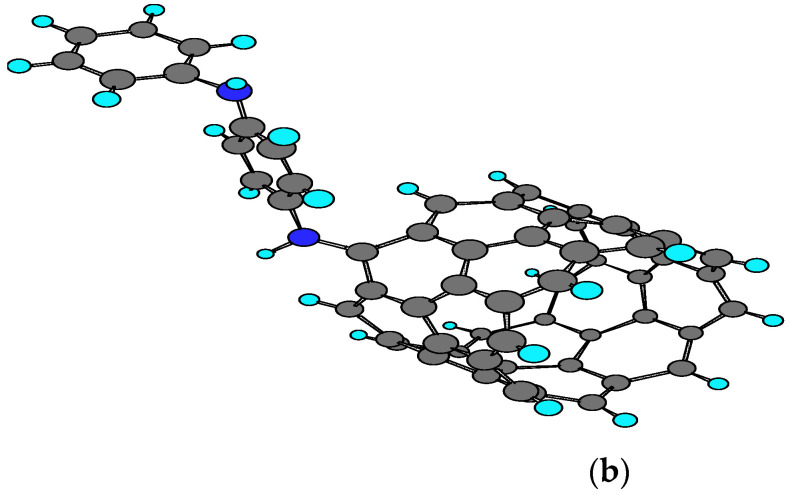
Modeling structure of PANI (**a**), and of the D-PAN/SWCNT composite (**b**).

To theoretically support the modifications that occur after doping and the insertion of SWCNTs, we use the already proposed modeling structures (Scheme 2) [29]. In fact, for the PANI, justifications are based on DFT calculations of the HOMO-LUMO energy difference in both the pure and doped states as a function of the chain length (n: monomer number). The properties of the polymer with infinite chain length are obtained by extrapolation (1/n → 0) [68]. Principally, it has been found that these energetic levels at the infinite length take the values of 3.62 eV and 2.29 eV, which are close to the experimental results.

The modeling structure of the nano-composite is, however, evidenced via the selection of PANI reactive sites based on atomic charge modification, spin density variation and theoretical infrared spectra. The results demonstrate that reactive sites for the grafting of SWCNTs are located around nitrogen atoms. A similar result has already been found in a systematic SERS and FTIR experimental study, but, in that case, the study was conducted with pure PANI [69]. It is important to note that fragments resulting from non-covalent bonding may also be present on the final structure. The preponderance of non-covalent or covalent interacting fragments is strictly dependent on the strength of the SWCNTs’ cohesive Van der Waals force, which inhibits the homogeneous dispersion of carbon nanotubes [70,71].

**Figure 11:**
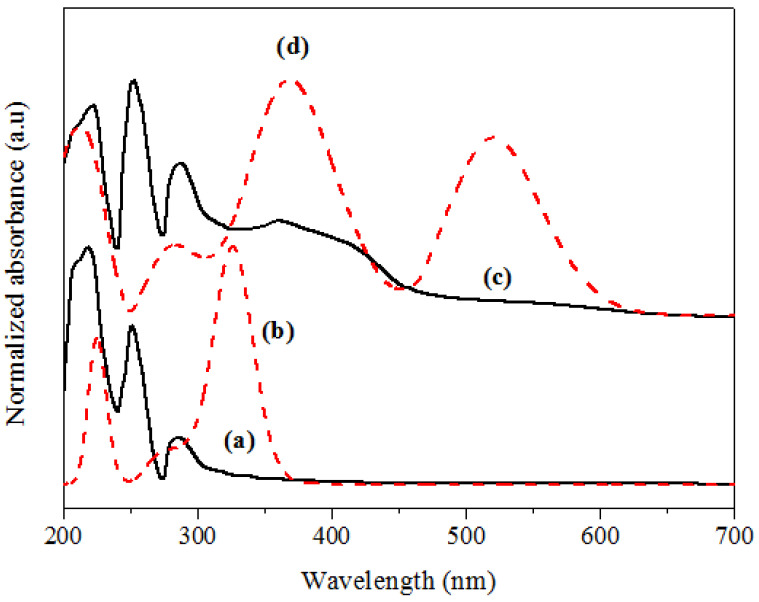
Optical absorption spectra of PANI—(**a**): experimental; (**b**): theoretical and those of doped-Pani/SWCNTs nano-composite; (**c**): experimental; (**d**): theoretical.

The above-presented modeling structures are used to calculate the optical absorption spectra of both PANI to PANI/SWCNTs composite, as presented in Figure 11. 

Referring to the experimental results, the same optical transitions are found and there is a decrease in the optical band gap after doping and after adding SWCNTs. It is suggested that theoretical band intensities are more pronounced than those obtained experimentally, due to the fact that calculations are carried out on a modeling structure with a higher concentration of SWCNTs than the real sample. In addition, the real sample presents additional interchain interactions, since calculations are expected to be performed with isolated fragments. When SWCNTs are added, the theoretical optical absorption spectrum is considerably affected. Particularly, new absorption band appears at λmax = 535 nm, giving rise to an absorption edge of 1.7 eV. It is believed that this new band is attributed to charge transfer creation [57]. If there is compatibility between the diffusion length and the nano-junction size, the created electron–hole pairs after photo-excitation can be easily transferred to SWCNTs, as already reported for other polymer/SWCNT nano-composites [72]. This charge transfer is the major parameter leading to improved photovoltaic characteristics [73]. Based on the PL results, the band created in the absorption regime after the insertion of SWCNTs is not luminescent. From these findings, it was concluded that after photo-exciton, the separation process is favored rather than the recombination via the electric field created by the resulting hetero-nano-junctions.

## 4. Conclusions

The correlation between both the experimental and theoretical results makes it possible to conclude that the PANI doping process with the sulfonic acid has been successfully conducted. The easy doping process permits the advantageous modification of the properties of the pure PANI. The band gap of PANI was reduced from 3.75 eV to reach 2.63 eV. Moreover, the localized states had a bandwidth of nearly 380 meV, and these were created with an energy level of 1.62 eV. On the other hand, the functionalization process of the doped PANI with SWCNTs involves grafting between the nitrogen atoms and the side wall. The resulting interpenetrating network presents an optical gap in the range of 2.28 eV. The nano-hetero-junctions give rise to the formation of localized states that result from the charge transfer with an Urbach energy of 80 meV. The stationary photoluminescence measurements make it possible to affirm that these localized states do not provide radiative recombination processes. The luminescence is strongly quenched after either doping or the insertion of SWCNTs. The correlations found in the experimental results provide evidence that the created nano-junction leads to the charge transfer, where SWCNTs play the role of dissociation sites of the exciton constituents. Due to the good compatibility with the solar spectrum in the absorption process, the resulting interpenetrating network can be used as an active layer for photovoltaic solar cells and a photocatalyst for advanced oxidation processes.

## Figures and Tables

**Table 1:** FTIR vibrational modes of PANI in both pure and doped states.

Frequency (cm^−1^)		Intensity Ratio (I_doped_/I_pure_)	Assignment
Neutralpure	Doped		
621	619	0.98	Torsion C-N outside the plane
1023	1020	1.02	C-H deformation
1132	1126	1.00	Benzene ring vibration
1257	1255	0.65	C-N of the benzene ring vibration
1382	1386	0.70	CH in the plane switching
1502	1505	1.09	Ring deformation
1554	1555	3.76	Quinoid aromatic C=C vibration
1656	1660	0.78	Benzene aromatic C=C vibration
2877	2866	0.81	Symmetric C-H stretching
2962	2941	0.91	N-H deformation
3643	3646	0.83	N-H stretching

## Data Availability

The data that supporting the reported results of this study will be made available by the authors upon request.
